# A patient with glycogen storage disease type Ib presenting with acute myeloid leukemia (AML) bearing monosomy 7 and translocation t(3;8)(q26;q24) after 14 years of treatment with granulocyte colony-stimulating factor (G-CSF): A case report

**DOI:** 10.1186/1752-1947-2-319

**Published:** 2008-09-30

**Authors:** Thomas Schroeder, Barbara Hildebrandt, Ertan Mayatepek, Ulrich Germing, Rainer Haas

**Affiliations:** 1 Department of Hematology, Oncology and Clinical Immunology, Heinrich-Heine-University, Moorenstr 5, 40225 Duesseldorf, Germany; 2 Institute for Human Genetics and Anthropology, Heinrich-Heine-University, Moorenstr. 5, 40225 Duesseldorf, Germany; 3 Department of General Pediatrics, Heinrich-Heine-University, Moorenstr. 5, 40225 Duesseldorf, Germany

## Abstract

**Introduction:**

Glycogen storage disease type Ib is an autosomal recessive transmitted disorder of glycogen metabolism caused by mutations in the glucose-6-phosphate translocase gene on chromosome 11q23 and leads to disturbed glycogenolysis as well as gluconeogenesis. Besides hepatomegaly, growth retardation, hypoglycemia, hyperlactatemia, hyperuricemia and hyperlipidemia, patients suffer from neutropenia associated with functional defects predisposing for severe infections. In order to attenuate these complications, long-term treatment with granulocyte colony-stimulating factor is common but this is associated with an increased risk for acute myeloid leukemia or myelodysplastic syndromes in patients with inherited bone marrow failures such as severe congenital neutropenia. Onset of these myeloid malignancies is linked to cytogenetic aberrations involving chromosome 7. In addition, granulocyte colony-stimulating factor is known to stimulate proliferation of monosomy 7 cells *in vitro*. To our knowledge, we report for the first time a case report of a patient with glycogen storage disease type Ib, who developed acute myeloid leukemia with a classical monosomy 7 and acute myeloid leukemia-associated translocation t(3;8)(q26;q24) after 14 years of continuous treatment with granulocyte colony-stimulating factor.

**Case presentation:**

A 28-year-old Turkish man with glycogen storage disease type Ib was admitted to our department because of dyspnea and increasing fatigue. He also presented with gum bleeding, bone pain in his legs, night sweats, recurrent episodes of fever with temperatures up to 39°C and hepatosplenomegaly.

A blood count taken on the day of admission showed pancytopenia and a differential count displayed 30% blasts. A bone marrow biopsy was taken which showed a hypercellular marrow with dysplastic features of all three cell lines, while blast count was 20%. Classical cytogenetic analyses as well as fluorescence in situ hybridization showed a monosomy 7 with a translocation t(3;8)(q26;q24). Based on these findings, the diagnosis of acute myeloid leukemia was made.

**Conclusion:**

Our observations suggest that bone marrow examinations including cytogenetic analysis should be carried out on a regular basis in patients with glycogen storage disease type Ib who are on long-term treatment with granulocyte colony-stimulating factor for severe neutropenia, since this treatment might also contribute to an increased risk for acute myeloid leukemia or myelodysplastic syndromes.

## Introduction

Glycogen storage disease type Ib (GSD-Ib), one of over 12 inherited metabolic disorders of glycogen metabolism, is an autosomal recessive disease caused by mutations in the glucose-6-phosphate translocase (G6PT) gene on chromosome 11q23. As a result of the G6PT deficiency, glycogenolysis as well as gluconeogenesis is disturbed. Patients suffer from hepatomegaly, growth retardation, hypoglycemia, hyperlactatemia, hyperuricemia and hyperlipidemia. The disease is further characterized by neutropenia associated with functional defects predisposing for severe infections including perioral and perianal ulcers as well as inflammatory bowel disease. In order to attenuate these complications, long-term treatment with granulocyte colony-stimulating factor (G-CSF) is common [1].

## Case presentation

We report the case of a 28-year-old Turkish man with a GSD-Ib who received treatment with G-CSF for 14 years because of disease-associated neutropenia. He has now developed acute myeloid leukemia (AML) with monosomy 7 and translocation t(3;8)(q26;q24). Admission to our hospital was required because of dyspnea and increasing fatigue. He also presented with gum bleeding, bone pain in his legs, night sweats, and recurrent episodes of fever with temperatures up to 39°C.

At the age of 1 year, a liver biopsy including enzyme function tests, which was performed because of hepatomegaly, recurrent hypoglycemia, growth retardation and bacterial infections of the upper respiratory system, confirmed the diagnosis GSD-Ib. As accessible in 1999, a mutational analysis of peripheral blood cells showed a homozygous mutation of exon 8 of the G6PT-locus (GPTL c.1211 del CT/c.1211 del CT) confirming this diagnosis. In the past, he had experienced recurrent episodes of hypoglycemia and hyperlactatemia associated with severe metabolic decompensations often requiring hospitalization. In order to prevent these metabolic disturbances, a special diet consisting of regular, carbohydrate-rich meals every 2 hours and ingestion of uncooked cornstarch twice per night was necessary. Because of relapsing oral and perianal aphthous ulcers as a result of severe neutropenia and neutrophilic dysfunction as indicated by a decreased deoxyglucose uptake, he received either filgrastim or lenograstim from the age of 15 years at different dose levels. In detail, cumulative duration of G-CSF therapy was 12.84 years and time-averaged dose was 2.83 μg/kg/day, leading to a total cumulative dose 13,729 μg/kg. An increase in white blood cell (WBC) count from baseline values of 3200/μl with a median of 14% neutrophils to a WBC count of 4245/μl with a median of 38% neutrophils could be achieved. The rise in neutrophil counts led to a substantial reduction in infectious complications. Bone marrow examinations were performed four times since the beginning of G-CSF treatment. The last examination was carried out at the age of 22 years showing no signs of leukemic transformation.

The patient is retarded in growth with a size of 160 cm and a body weight of 50 kg. The color of his skin is pale and he has hardly any secondary body hair. We found a few aphthous ulcers scattered in the oral cavity and mucosa bleeding. As confirmed by abdominal sonography, the patient had hepatosplenomegaly (liver 124 mm craniocaudal diameter × 148 mm dorsoventral diameter in medioclavicular line, spleen length 138 mm × depth 57 mm). Blood count taken at the day of admission showed pancytopenia with a WBC count of 2.000/μl, reduced hemoglobin of 4.2 g/dl with normal erythrocyte indices and a platelet count of 71 × 10^3^/μl. Differential count was as follows: 62% lymphocytes, 4% monocytes, 4% mature neutrophilic granulocytes and 30% blasts. A first bone marrow biopsy was taken showing a hypercellular marrow with dysplastic features of all three cell lines, while blast count was 20%. This result was confirmed by flow cytometry analysis with 30% CD34 positive cells.

**Figure 1:**
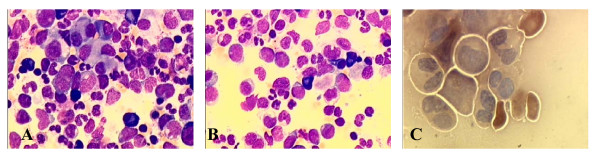
**Cytomorphological bone marrow examination performed on the day of admission to our hospital showing a cellular marrow with dysplastic features in all three cell lines and elevated blast count of about 20%**. A) This image displays micromegakaryocytes and Pseudo-Pelger cells as dysplastic features of the granulopoiesis. B) This image shows an increased blast count of 20% and Pseudo-Pelger cells. C) This image demonstrates the total deficiency of myeloperoxidase.

**Figure 2:**
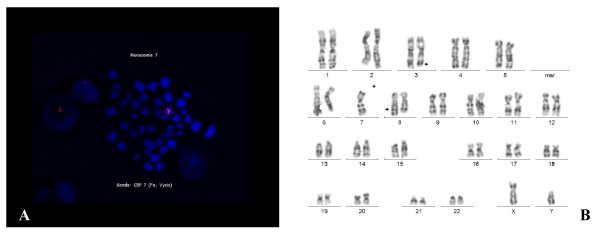
**Cytogenetic analysis of bone marrow cells performed on the day of admission to our hospital demonstrating monosomy 7 and an accessorial translocation t (3;8)**. A) This image displays detection of monosomy 7 by fluorescence in-situ hybridization using a CEP 7 probe (Vysis, USA). Monosomy 7 was found in 78.5% of the examined metaphases. B) This image shows classical cytogenetic banding of a representative metaphase of the patient with glycogen storage disease type Ib. In total, 34 metaphases have been analyzed demonstrating a monosomy 7 in 33 metaphases and a translocation t (3;8)(q26;q24).

The patient received four units of packed erythrocytes, which led to an increase in hemoglobin concentration to 10.1 g/dl. Empiric antimicrobial treatment was started because of recurrent fever and rising serum levels of the C-reactive protein. Despite X-ray examination of his thorax, laboratory and microbial analysis of urine and feces and daily physical examination, no source of infection could be found. Since the serum level of procalcitonin as a possible marker for an infection was normal, we interpreted the fever as disease-related. As a consequence of the discontinuation of G-CSF administration, the leukocyte count declined to 800/μl, while the proportion of blasts in the peripheral blood was 4%. The rest of the white blood cells were lymphocytes without any mature neutrophils. The platelet count decreased to 36.000/μl with an ongoing need for red blood cell transfusions. A second bone marrow biopsy was performed still showing an elevated blast count of 21% with the same features of dysplasia (Figure 1). Classical cytogenetic analyses showed a monosomy 7 in 33 of 34 analyzed metaphases with a translocation t(3;8)(q26;q24). Monosomy 7 as well as translocation t(3;8)(q26;q24) was confirmed by fluorescence in situ hybridization (FISH) in 78.5% of the examined metaphases (Figure 2). Based on these findings, the diagnosis of an acute myeloid leukemia with multilineage dysplasia with a typical monosomy 7 and translocation t(3;8)(q26;24) was made.

In the light of the increased risk profile of this patient due to his metabolic disorder and the low response rate to conventional chemotherapy of patients with chromosome 7 abnormalities, we administered a first course of DNA methyl transferase inhibitor 5-azacytidine (151 mg absolute subcutaneously daily for 5 days). This decision was made because chromosome 7 alterations predict response to treatment with this drug in patients with myelodysplastic syndromes (MDS) or AML [2]. At the time of writing this report, 100 days after the beginning of the first course of the epigenetic therapy, the patient is still alive. Transfusion-dependent pancytopenia persists, which is in line with prior studies showing a response to 5-azacytidine after 3 or 4 cycles [2]. Administration of a second course of 5-azacytidine was avoided by infectious complications until now. Because patients with chromosome 7 abnormalities have a poor prognosis, the use of myeloablative conditioning therapy allogeneic blood stem cell transplantation with his Human Leukocyte Antigen (HLA) identical twin sister is envisaged for the patient.

## Discussion

As in our patient, neutropenia and neutrophilic dysfunction are characteristic findings in patients with GSD-Ib leading to an increased risk of infectious diseases. Some of the molecular mechanisms underlying the neutrophilic dysfunctions in patients with GSD-Ib were unraveled by Kim *et al. *[3]. For instance, neutrophil granulocytes exhibit impaired respiratory burst as well as impaired chemotaxis and calcium flux. The neutrophils also have an increased preponderance for apoptosis which contributes to the development of neutropenia. The major reason for these disturbances is the G6PT-defiency in the endoplasmic reticulum (ER) membrane of neutrophils resulting in a lack of glucose and increased oxidative stress [3]. The results of our genome-wide expression data on CD34+ cells from bone marrow of normal donors (data not shown) suggest that G6PT is normally expressed in hematopoietic stem and progenitor cells. A G6PT-deficiency in the CD34+ progenitor might therefore be of functional relevance, in particular for the regular differentiation along the myeloid pathway while erythropoiesis and thrombopoiesis are not affected.

There are two case histories in the literature reporting on patients with GSD-Ib who had developed AML. One of them received G-CSF over a period of 6 years, while the other did not receive the growth factor. Cytogenetic analysis in the patient being treated with G-CSF showed a normal 46 XX karyotype in 3 cells and a 47 XX +10 karyotype in 1 cell [4,5]. Thus, considering the low incidence of GSD-1b, neither an increased intrinsic risk for AML nor an association between G-CSF treatment and evolution of myeloid neoplasms has been noted so far [6].

With regard to neutropenia, we will place this case in the context of other inherited bone marrow failures with isolated neutropenias such as severe congenital neutropenia (SCN) or Shwachman-Diamond syndrome (SDS). These diseases are associated with an enhanced risk for AML or MDS [7]. In addition to this intrinsic risk, the likelihood of developing myeloid neoplasms may also increase as a result of long-term treatment with G-CSF as shown by the data from the French Severe Chronic Neutropenia Study Group and the Severe Chronic Neutropenia International Registry [8,9]. Onset of AML or MDS in these patients is linked to cytogenetic aberrations involving chromosome 7 in up to 76% of patients with SCN and 66% of patients with SDS, respectively. Long-term treatment with G-CSF is also associated with evolution of MDS in patients with aplastic anemia involving monosomy 7 in up to 58% of the patients [6,7]. In the light of these findings, it is interesting to note that G-CSF is known to stimulate proliferation of monosomy 7 cells *in vitro *[10].

With regard to GSD-Ib, Donadieu *et al. *reported on a survey of 15 patients who were treated with G-CSF because of disease-related neutropenia [8]. In this context, it is worth noting that in our patient, the total cumulative duration of G-CSF therapy (12.84 years) was much longer than in the group reported by Donadieu *et al. *(median 2.8 years, range 0.1 to 8.8 years) whereas the time-averaged dose and total cumulative dose were comparable. Within this group of patients, there was no case of AML and no observations with regard to clonogenic evolution or structural or numeric aberrations of chromosome 7 [8].

Interestingly in our patient, at the age of 22 years, a cytogenetic analysis of bone marrow cells showed a monosomy 7 in 4.8% of the metaphases. This proportion was estimated under cut-off and considered as pathologically irrelevant. In the light of the actual finding with 78.5% of the metaphases bearing the monosomy 7, we conclude that the treatment with G-CSF might have contributed to the gradual expansion of the monosomy 7 clone over time. This view is supported by the finding of the translocation t(3;8)(q26;24) which had not been found on the occasion of the examination at the age of 22 years. This reciprocal translocation is known to lead to an aberrant expression of the proto-oncogene EVI1 and has been implicated to be a pathophysically relevant aberration for the development of an AML [11]. Thus, it might be postulated that, during the expansion of the hematopoietic stem cell clone bearing the monosomy 7 favored by continuous stimulation with G-CSF, the additional occurrence of the translocation t(3;8)(q26;24) was an important step for leukemogenesis.

## Conclusion

As a result of our observations, we suggest that bone marrow examinations including cytogenetic analysis should be carried out on a regular basis in patients with GSD-Ib who are on long-term treatment with G-CSF for severe neutropenia, since G-CSF might also contribute to the development of myeloid malignancies in these patients.

## Competing interests

The authors declare that they have no competing interests.

## Authors' contributions

TS initiated the report, collected and analyzed the patient data and undertook the majority of the writing of the manuscript. BH performed cytogenetic analyses and was involved in drafting the manuscript. UG performed the cytomorphological analysis of bone marrow, made contributions to the conception and design of the report and revised it critically for important intellectual content. EM made contributions to the conception and design of the report and revised it critically for important intellectual content. RH initiated the report, made contributions to the conception and design of the report and revised it critically for important intellectual content. All authors read and approved the final manuscript.

## Consent

Written informed consent was obtained from the patient for publication of this case report and any accompanying images. A copy of the written consent is available for review by the Editor-in-Chief of this journal.
